# Factors Contributing to Persistent Frequent Attendance in Primary Care Among the Oldest Old: Longitudinal Evidence From the AgeCoDe-AgeQualiDe Study

**DOI:** 10.3389/fmed.2022.815419

**Published:** 2022-03-21

**Authors:** Elżbieta W. Buczak-Stec, André Hajek, Hendrik van den Bussche, Marion Eisele, Anke Oey, Birgitt Wiese, Siegfried Weyerer, Jochen Werle, Angela Fuchs, Michael Pentzek, Melanie Luppa, Margit Löbner, Dagmar Weeg, Edelgard Mösch, Kathrin Heser, Michael Wagner, Steffi G. Riedel-Heller, Wolfgang Maier, Martin Scherer, Hans-Helmut König

**Affiliations:** ^1^Department of Health Economics and Health Services Research, Hamburg Center for Health Economics, University Medical Center Hamburg-Eppendorf, Hamburg, Germany; ^2^Department of General Practice and Primary Medical Care, Center for Psychosocial Medicine, University Medical Center Hamburg-Eppendorf, Hamburg, Germany; ^3^Hannover Medical School, Institute of General Practice, Hanover, Germany; ^4^Central Institute of Mental Health, Medical Faculty Mannheim/Heidelberg University, Mannheim, Germany; ^5^Medical Faculty, Institute of General Practice, Heinrich-Heine-University Düsseldorf, Düsseldorf, Germany; ^6^Institute of Social Medicine, Occupational Health and Public Health, University of Leipzig, Leipzig, Germany; ^7^Department of Psychiatry, Technical University of Munich, Munich, Germany; ^8^Department of Neurodegenerative Diseases and Geriatric Psychiatry, University Hospital Bonn, Bonn, Germany; ^9^German Center for Neurodegenerative Diseases (DZNE), Bonn, Germany

**Keywords:** aged, 80 and over, general practitioners (GP), health care utilization, health services needs and demand, primary health care, persistent frequent attendance, frequent attender

## Abstract

**Objective:**

Since there is a lack of longitudinal studies in this area, our aim was to identify the determinants of persistent frequent attendance in primary care among the oldest old in Germany.

**Methods:**

Longitudinal data (follow-up wave 7–9) were taken from the multicenter prospective cohort “Study on needs, health service use, costs, and health-related quality of life in a large sample of oldest-old primary care patients (85+)” (AgeQualiDe), covering primary care patients ≥ 85 years (FU7 *n* = 741, mean age 88.9 years (SD 2.9; 85–100)). Persistent frequent attenders of general practitioner (GP) services (the patients in the top decile of the number of GP consultations in two or more consecutive waves) were our main outcome of interest. Logistic random-effects models were used.

**Results:**

Our analysis included 1,891 observations (766 individuals). Across three waves, we identified 56 persistent frequent attenders. Results of random-effects logistic regressions showed that the odds of being persistent frequent attender were higher for widowed individuals (OR = 4.57; 95% CI [1.07–19.45]). Moreover, a one-point increase in the frailty score and having one more chronic condition increased the odds of being a persistent frequent attender by 68% (OR =1.68; 95% CI [1.05–2.69]) and 23% (OR=1.23, 95% CI [1.05–1.44]), respectively.

**Conclusion:**

Our study stressed the longitudinal association between frailty and widowhood as well as chronic diseases and persistent frequent attendance among the oldest old in Germany.

## Introduction

A large number of older individuals have regular contacts with their general practitioner (GP), and the number of visits commonly reflects their increasing morbidity and health problems. However, some of those patients may overuse healthcare services. Moreover, for some patients, the underlying cause of the visit may not be attributed to medical reasons. It is well known that so-called frequent attenders in primary care (particularly individuals in late life) produce a great burden for health care systems since this rather small group causes a large proportion of visits to the GP (1). Additionally, frequent attendance can also lead to feelings of stress, frustration, and burnout among physicians in different countries (2–5). This in turn can negatively affect both patient and doctor satisfaction (6–10). For the aforementioned reasons, knowledge about the factors leading to persistent frequent attendance among individuals in late life is of great importance.

A previous systematic review predominantly identified several cross-sectional studies that examined the determinants of frequent attenders in primary care among individuals in the higher age group (11). These cross-sectional studies mainly revealed an association between frequent attendance and physical illnesses. In addition, a recent systematic review (12) exclusively including longitudinal studies showed that comparable findings, e.g., physical functioning, physical illnesses, and unemployment were associated with persistent frequent attendance. Furthermore, this study emphasized the importance of investigating the determinants of persistent frequent attendance. Particularly, persistent frequent attendance is important for the health care system (13). It may indicate that the healthcare needs of the patient are not satisfied or not met. Beyond need factors (such as health-related factors), other factors may play a role in persistent frequent attendance e.g., psychosocial factors such as social isolation (or personality-related factors). Knowing the determinants of persistent frequent attendance in primary care could be useful from a clinical perspective and reduce the economic burden associated with it (12).

To put our findings into context, some important characteristics of the German health care system are worth describing. First, health insurance is compulsory in Germany. Additionally, most of the individuals (about 90%) are insured by social statutory health insurance (SHI) funds, whereas the remaining 10%, mainly covering civil servants, employees above a certain income threshold, and self-employed individuals, are insured by private health insurance (PHI). Most expenses of outpatient treatment are covered by both SHI and PHI. Furthermore, it may be worth noting that all individuals have access to outpatient specialist services without a referral from GPs. Moreover, waiting times for outpatient physician visits are rather short when compared internationally (14), indicating that in general individuals have a rather good access to health care services in Germany. Additional details are presented elsewhere (15).

Since there is presumably a large increase in the number of individuals 85 years and over (“oldest old”) in the next decades and individuals in this age bracket have high health expenditure per capita, knowledge about the drivers of persistent frequent attendance in primary care among the oldest old is of importance. However, we only identified one cross-sectional study explicitly focusing on frequent attendance in primary care, but not persistent frequent attendance, among the oldest old (16). This study showed that in this age group, frequent attendance was mainly associated with worse physical health (in terms of more physical illnesses and worse physical functioning). However, there is a lack of longitudinal studies focusing on the factors leading to persistent frequent attendance in primary care among the oldest old. Compared to cross-sectional studies, such longitudinal studies are necessary to gain further insights into the factors leading to persistent frequent attendance. Moreover, using longitudinal data can assist in obtaining more precise and consistent estimates. Therefore, this longitudinal study aimed to identify the determinants of persistent frequent attendance in primary care among the oldest old in Germany.

In turn, knowledge about the factors leading to persistent frequent attendance could improve health care management and avoid mis- or overuse of primary care. In accordance with previous research regarding frequent attendance, we particularly hypothesize that health-related factors such as frailty are associated with persistent frequent attendance among the oldest old (12). Beyond that, and particularly among the oldest old, social factors (e.g., social isolation) may also be important for persistent frequent attendance because many friends and relatives are likely to be deceased already. Frequent GP visits may thus compensate for meeting friends and relatives. Hence, we assume that social isolation may be associated with persistent frequent attendance.

## Methods

### Sample

For this observational cohort study, we used longitudinal data from the Study on needs, health service use, costs, and health-related quality of life in a large sample of oldest-old primary care patients (85+) (AgeQualiDe). The AgeQualiDe study is a multicenter prospective cohort study that started in January 2014. Data from primary care patients aged 85 years and older were obtained from six research centers: Hamburg, Bonn, Düsseldorf, Leipzig, Mannheim, and Munich. It is an extension and continuation of the preceding large cohort study “German Study on Aging, Cognition, and Dementia in Primary Care Patients (AgeCoDe).” For more details describing the AgeCoDe/AgeQualiDe, please refer to the study by Luck et al. (17).

Briefly, AgeCoDe started in 2003/2004, with patients recruited *via* GP offices (at each of the six study centers, 19–29 GPs). To be eligible for the study in 2003, the participants had to meet a number of criteria (at baseline). Participants were regular patients of GPs, were aged at least 75 years, were not institutionalized, did not have dementia, and were not deaf or blind. Furthermore, they had sufficient knowledge of the German language and were able to provide consent to participate in the study. Individuals with severe conditions that the GP would classify as fatal within 3 months were excluded. The individuals or their proxies were interviewed by the trained staff. The interview took place at the patients' homes.

In this study, we used longitudinal data from follow-up 7 (FU7; *n* = 861) to follow-up 9 (FU9) due to data availability. The primary form of data collection was a standardized interview, and complementary assessments were conducted by specially trained interviewers (e.g., doctors or psychologists) at the patients' homes. Data were collected in 10-month intervals starting in 2014 (FU7) and ending in 2016 (FU9). At FU7, 861 individuals (*n* = 861) participated (from *n* = 955 who were still alive at FU7). Drop-out occurred primarily due to death or ill health. We restricted our analysis to respondents who provided information about GP visits and with no missing covariate data in at least one wave (FU7–FU9).

The AgeCoDe and the AgeQualiDe study were approved by the ethics committees of all participating study centers (approval numbers: Hamburg: OB/08/02, 2817/2007, MC-390/13; Bonn: 050/02; 174/02, 258/07, 369/13; Mannheim: 0226.4/2002, 2007-253E-MA, 2013-662 N-MA; Leipzig: 143/2002, 309/2007, 333-1318112013; Düsseldorf: 2079/2002, 2999/2008, 2999; and München: 713/02, 713/02 E) and complied with the ethical standards of the Declaration of Helsinki. All individuals gave written informed consent to participate in the study.

### Dependent Variables

Being a persistent frequent attender of GP services was our outcome of interest. To consider an individual as a persistent frequent attender, we first calculated the number of GP consultations. For this purpose, we used the survey question “Have you visited a GP or family doctor in the last 3 months? How often have you been to this doctor?” Following the definition used widely in other studies [as an overview please see the two systematic reviews regarding frequent attendance (11, 12)], frequent attenders in each wave were the patients in the top decile of the number of GP consultations (this represents 6 and more GP consultations within the past 3 months). Persistent frequent attenders were defined as patients who were frequent attenders in two or three consecutive waves. Nonpersistent frequent attenders were defined as all other GP patients.

### Independent Variables

The covariates used in the analysis were selected on the basis of a comprehensive systematic review (11, 12) and research on persistent frequent attendance (18–21). We also considered Andersen's model of health services use, which segregates factors into physical needs factors, predisposing characteristics, and enabling resources (22).

First, in our analysis, we controlled for socio-demographic variables *(predisposing characteristics)*. We included information on sex, age, widowhood status (widowed, nonwidowed), and the educational level [elementary, secondary, and higher education according to Comparative Analysis of Social Mobility in Industrial Nations (CASMIN) (23)]. In our analysis, we dichotomized the responses into elementary education and other (secondary and higher education). As persistent frequent attendance may depend on the type of health care insurance *(enabling resources)*, we also included the patient's insurance status in our analysis (dichotomized into private and statutory health insurance). Moreover, since socially isolated individuals may visit their GP more often, we also controlled for social isolation. To measure social isolation we used the valid 6-item version of the Lubben Social Network Scale (LSNS-6) (24). The scale ranges from 0–30 (the higher the score, the higher the perceived support). Scores below 13 indicate social isolation (24).

We also controlled for a set of variables describing physical and mental health status *(need factors)*. Cognitive impairment was measured using the Global Deterioration Scale. The scale ranges from 1 (indicating no impairment) to 7 (indicating severe cognitive impairment) (25). Additionally, we also controlled for subjective memory impairment. To assess subjective memory impairment, individuals were asked “Do you feel your memory is getting worse?” (yes; no). We used the 15-item version of the Geriatric Depression Scale to operationalize depressive symptoms (26). The Geriatric Depression Scale is a validated scale for older populations, it ranges from 0 to 15 points, with higher values corresponding to more depressive symptoms (27). A score of 6 and more indicates depression (28, 29).

Frailty was assessed using the Canadian Study of Health and Aging (CSHA) Clinical Frailty Scale (CFS). The CFS is a clinically based instrument that summarizes the outcomes of the comprehensive geriatric assessment (30). The scale ranges from 1 = “very fit” (robust, active, energetic, and well-motivated; these people commonly exercise regularly and are in the fittest group for their age) to 7 = “severely frail” (completely dependent on others for the activities of daily living or terminally ill) (31).

We used the visual analog scale of the EQ-5D (EQ-VAS) (32) to assess health-related quality of life (HRQoL). Participants were asked to rate their current HRQoL on the EQ-VAS ranging from 0–100, with 0 indicating the worst imaginable health state and 100 indicating the best imaginable health state.

In the first model, we controlled for all above-listed variables. In another model, we controlled for functioning instead of frailty, since frailty and functioning were quite strongly correlated in our dataset. To assess functioning we used the Barthel index. This index ranges from 0 to 100, with lower values indicating worse functioning (33). In an additional model, we also controlled for the number of chronic diseases. Information on the presence of 35 chronic diseases (e.g., diabetes, stroke, Parkinson's, COPD, and epilepsy) was provided by a patient's GP. We used a count score of these variables to indicate the number of chronic diseases. Chronic diseases were not included in the main model due to the missing values (since by FU9 many doctors had already retired and could no longer collect data).

### Statistical Analysis

In order to study the factors contributing to the persistent frequent attendance, we used logistic random-effects models with participant-level random effect [29]. These models are appropriate for repeated measures data (clustered dichotomous responses). Random effects are selected to account for specific factors which are expected to cause random variation in the coefficients (e.g., within subject variation in repeated measures) (34). We presented the results as subject-specific (conditional) odds ratios with 95% confidence intervals. All models were adjusted for the variables listed above. It should be noted that all determinants are included from FU7 to FU9. Listwise deletion was used to handle missing data in regression analysis. The criterion for statistical significance was set at *p* < 0.05. For analysis, we used Stata 16.0 (Stata-Corp, College Station, Texas, USA).

## Results

### Descriptive Statistics

In total, across three waves (FU7-FU9), our analysis included 1,891 observations (involving 766 individuals). Altogether, we identified 56 persistent frequent attenders (individuals who were frequent attenders in two or more consecutive waves; 40 in FU7 and an additional 16 in FU8).

Descriptive statistics for the FU7 data are reported in Table 1. At FU7, information without missing covariates were available from 741 individuals (499 women and 242 men). The average age was 88.9 (SD 2.9) years (85–100 years). In FU7, we identified 40 individuals (5.4%) who were persistent frequent attenders. Compared to nonpersistent frequent attenders, persistent frequent attenders had more diagnosed conditions (median value of chronic diseases was 6 vs. 7.5), had higher frailty levels [4.6 (1.4) vs. 3.9 (1.4)], and their functioning was worse [82.8 (22.5) vs. 92.3 (13.4)]. Moreover, persistent frequent attenders differed from nonpersistent frequent attenders in terms of widowhood status (82.5% vs. 62.2%). The differences with regard to e.g., age, sex, education, HRQoL, and cognitive impairment were not statistically significant (Table 1).

**Table 1:** Descriptive characteristics of study cohort at baseline (FU7) - persistent frequent attender and nonpersistent frequent attender.

**Mean (SD); range / n (%)**	**Non-persistent frequent attender**	**Persistent frequent attender**	***p*-value**
	***n =* 701**	***n =* 40**	
Age	88.85 (2.92); 85-100	89.72 (3.15) 86-97	0.07
Sex			0.71
Female	471 (67.2%)	28 (70.0%)	
Male	230 (32.8%)	12 (30.0%)	
Widowhood status			0.01
Widowed	436 (62.2%)	33 (82.5%)	
Non-widowed	265 (37.8%)	7 (17.5%)	
Education			0.49
Primary	390 (55.6%)	20 (50.0%)	
Secondary and high	311 (44.4%)	20 (50.0%)	
Health insurance			0.23
Private health insurance	65 (9.3%)	6 (15.0%)	
Statutory health insurance	636 (90.7%)	34 (85.0%)	
Depressive symptoms (Geriatric Depression Scale)	2.73 (2.59); 0–15	2.95 (2.23); 0–9	0.61
Cognitive decline (Global Deterioration Scale)	1.95 (0.93); 1–5	2.23 (1.19); 1–5	0.07
Subjective memory impairment:			0.10
No	266 (37.9%)	10 (25.0%)	
Yes	435 (62.1%)	30 (75.0%)	
Social isolation:			0.14
No (LSNS ≥ 13)	415 (59.2%)	19 (47.5%)	
Yes (LSNS ≤ 12)	286 (40.8%)	21 (52.5%)	
Health-related quality of life (EQ-VAS)	62.79 (18.77); 5-100	59.13 (16.68); 0-90	0.23
Frailty (CSHA-CFS)	3.85 (1.42); 1-7	4.60 (1.37); 2-7	<0.001
Functioning (Barthel-Index)	92.29 (13.42); 15-100	82.75 (22.45); 5-100	<0.001
Number of chronic diseases	6.06 (3.02); 0-18	7.97 (3.32); 2-15	<0.001
Median (Q1, Q3)	6 (4, 8)	7.5 (5, 10)	

*AgeQualiDe - Study on needs, health service use, costs and health-related quality of life in a large sample of oldest-old primary care patients (85+)*.

SD, standard deviation; p-values are based on chi-square or Fisher exact tests for categorical variables and on independent t-tests for continuous variables; Q1, Q3 - 25th and 75th percentiles. For individuals with severe cognitive decline with MMSE (Mini-Mental Status Test) score <19^*^, e.g., health-related quality of life was not drawn; for individuals with Global Deterioration Scale ≥4, e.g., number of GP visits, social isolation were not drawn; standard deviation in parenthesis; Education - according to the new CASMIN educational classification (47); Social isolation - 6-item version of the Lubben Social Network Scale (LSNS-6); score from 0 to 30, scores less than 13 points denote social isolation (24). Depression - short version of the Geriatric Depression Scale, which contains 15 items (GDS-SF); (26). Frailty - Canadian Study of Health and Aging (CSHA) Clinical Frailty Scale (CFS) (1 = “very fit to 7 = “severely frail”) (31). Functioning - Barthel Index ranging from 0 to 100, higher values indicating better functioning (33); Cognitive decline - Global Deterioration Scale (1 = “no impairment” to 7 = ”severe cognitive impairment”) (25).

### Regression Analysis

The results of random-effects logistic regressions are shown in Table 2. Regression showed (Model 1) that conditional (subject-specific) odds of being persistent frequent attenders were higher for widowed individuals compared to nonwidowed individuals (OR = 3.65; 95% CI [1.04-12.83]). Moreover, a one-point increase in the frailty score increased the odds of being a persistent frequent attender by 66% (OR = 1.66; 95% CI [1.09–2.53]). Furthermore, compared to individuals with a private health insurance, individuals with statutory health insurance had lower odds of being a persistent frequent attender (OR = 0.18, 95%CI [0.04–0.76]).

**Table 2:** Determinants of persistent frequent attenders among the oldest old (85–100 years old, *n* = 766). Results of the random-effects logistic regression.

	**Model 1**		**Model 2**		**Model 3**		**Model 4**	
**Variables**	**OR**	**95%CI**	**OR**	**95%CI**	**OR**	**95%CI**	**OR**	**95%CI**
								
Male	1.01	(0.29–3.54)	1.58	(0.39–6.38)	1.05	(0.29–3.73)	1.85	(0.44–7.73)
Age	1.05	(0.88–1.26)	1.01	(0.82–1.24)	1.05	(0.87–1.25)	0.98	(0.80–1.21)
Widowed (ref. non-widowed)	3.65^*^	(1.04–12.83)	4.57^*^	(1.07–19.45)	3.89^*^	(1.06–14.28)	6.94^*^	(1.53–31.55)
Education (ref. other)								
Primary	1.04	(0.35–3.14)	1.19	(0.34–4.15)	1.07	(0.35–3.23)	1.35	(0.38–4.78)
Health insurance (ref. private)								
Statutory health insurance	0.18^*^	(0.04–0.76)	0.57	(0.10–3.39)	0.18^*^	(0.04–0.81)	0.59	(0.10–3.65)
Depressive symptoms	1.06	(0.88–1.27)	1.05	(0.85–1.31)	1.01	(0.84–1.22)	0.98	(0.77–1.23)
Subjective memory impairment (ref. no)								
Yes	1.04	(0.40–2.66)	1.38	(0.45–4.22)	1.24	(0.48–3.23)	1.69	(0.53–5.41)
Social isolation	1.24	(0.47–3.24)	0.85	(0.28–2.53)	1.16	(0.44–3.10)	0.89	(0.29–2.73)
Health-related quality of life (EQ-VAS)	0.98	(0.96–1.01)	0.99	(0.96–1.02)	0.99	(0.96–1.01)	0.99	(0.96–1.02)
Frailty	1.66^*^	(1.09–2.53)	1.68^*^	(1.05–2.69)				
Cognitive decline	1.44	(0.90–2.31)	1.48	(0.84–2.60)	1.20	(0.74–1.94)	1.31	(0.73–2.35)
Number of chronic diseases			1.23^*^	(1.05–1.44)			1.19^*^	(1.01–1.41)
Functioning (Barthel-Index)					0.94^***^	(0.91–0.96)	0.93^***^	(0.90–0.97)
Random part	✓		✓		✓		✓	
Observations	1,891		1,420		1,892		1,421	
Number of individuals	766		618		766		618	

*AgeQualiDe - Study on needs, health service use, costs and health related quality of life in a large sample of oldest-old primary care patients (85+). Data collection baseline in 2014 and 2 follow-ups in a 10-months interval*.

*Odds ratios are reported, CI – 95% confidence intervals; Education - according to the new CASMIN educational classification [47]; Social isolation - 6-item version of Lubben Social Network Scale (LSNS-6); score from 0 to 30 [31]. Depression - short version of the Geriatric Depression Scale, which contains 15 items (GDS-SF) [24]. Functioning - Barthel Index ranging from 0 to 100, higher values indicating better functioning [33]; Cognitive decline - Global Deterioration Scale (1 = “no impairment” to 7 = ”severe cognitive impairment”) [23]; Frailty - Canadian Study of Health and Aging (CSHA) Clinical Frailty Scale (CFS) (1 = “very fit to 7 = “severely frail”) (31). Analysis was adjusted for follow-up wave; Social support - 6-item version of Lubben Social Network Scale (LSNS-6); score from 0 to 30 (highest social support) (24)*.

*** *p <0.001*,

**p <0.01,

* *p <0.05, + p <0.10*.

In the second model, we additionally controlled for the number of chronic conditions (Table 2, Model 2). The regression results additionally showed that having more chronic conditions increased the odds of being a persistent frequent attender (OR = 1.22, 95%CI [1.04–1.43]). In contrast, the association between insurance status and persistent frequent attendance was not significant anymore (OR = 0.58, 95%CI [0.10–3.42]).

In all models, no statistically significant association between being a persistent frequent attender and age, sex, depressive symptoms, social isolation, HRQoL, and cognitive decline were identified.

In another robustness check, we controlled for functioning instead of frailty (Model 3: without chronic conditions as covariate and Model 4: with chronic conditions as covariate). The results were the same in terms of significance and direction. In this case, better functioning reduced the odds of being a persistent frequent attender (e.g., Model 3, OR=0.94, 95%CI [0.91–0.96]).

## Discussion

Based on the longitudinal data from a large, multicentre prospective cohort study, this study aimed to clarify the determinants of persistent frequent attendance in primary care among the oldest old. Regressions showed that the likelihood of persistent frequent attendance increased with being widowed, higher frailty levels, and more chronic conditions. Our current longitudinal study adds to the very limited knowledge regarding the determinants of persistent frequent attendance in later life (12).

In general, our findings are somewhat difficult to compare with other longitudinal studies examining the determinants of frequent attendance in later life since previous longitudinal studies only partly focused on persistent frequent attendance (12). This should be acknowledged when comparing our results with previous longitudinal studies.

With regard to predisposing characteristics, we found an association between being widowed and a higher likelihood of persistent frequent attendance, which is in contrast to our previous work (16). Such differences may particularly be explained by differences in the design and analytical approach (cross-sectional vs. longitudinal regression models) as well as slight differences in the outcome (frequent attendance at one single point in time vs. persistent frequent attendance). To date, there is rather inconclusive evidence regarding marital status and frequent attendance (12, 19, 35–37). Our findings may be explained by the fact that widowed individuals may miss their contact with their deceased spouse. Persistent frequent attendance may, at least partially, replace this personal contact. Moreover, some recently widowed individuals who experience difficulties during this transition (38) may also more frequently seek help from their GPs. Another explanation may be that friends and relatives may often be worried about the health of these widowed individuals. Consequently, they may urge them to visit a GP regularly and frequently later in life. However, this is a hypothetical explanation that could be examined in future studies.

The other predisposing characteristics (i.e., age and sex) were not associated with persistent frequent attendance. In contrast, a recent systematic review showed that increasing age was frequently associated with a lower probability of becoming a frequent attender (12). These differences may be explained by differences in the outcome measure (frequent attendance vs. persistent frequent attendance in our study) and differences in the age groups (mainly older adults vs. oldest-old individuals in our study). Additionally, our study contributes to the inconclusive evidence on the association between sex and persistent frequent attendance (12).

With regard to enabling resources, individuals with statutory health insurance had lower odds of being a persistent frequent attender compared to individuals with a private health insurance. This may be mainly explained by supply-induced demand in healthcare in Germany. This is in line with previous research conducted in Germany (39). Furthermore, future research is required to examine the rarely investigated potential association between social isolation and persistent frequent attendance among the oldest old (12).

In accordance with a recent systematic review (12), we found an association between need factors (i.e., level of frailty and number of chronic conditions) and persistent frequent attendance. However, it should be noted that previous longitudinal studies mainly focused on self-rated health or physical functioning [e.g., (19, 21, 40)], but not on frailty. These findings appear very plausible since frailty and chronic conditions may indicate high healthcare needs (41), which could require continuous frequent GP visits. This may highlight the importance of frailty for persistent frequent attendance.

In contrast, our study did not identify an association between the other need factors (i.e., depressive symptoms, HRQoL, and cognitive decline) and persistent frequent attendance, in contrast to our own previous work (16). It should be noted that while our previous cross-sectional study focused on the association between depression (dichotomous) and frequent attendance, our current longitudinal study focused on the association between depressive symptoms (continuously measured) and persistent frequent attendance. Such differences in the variables (depression vs. depressive symptoms; frequent attendance at one single point in time vs. persistent frequent attendance) and differences in the analytical approach (i.e., cross-sectional regression models vs. longitudinal regression models) may explain such differences.

Our study thus adds to the inconclusive evidence regarding the link between mental health and frequent attendance (12). It seems that the country of origin of the study participants and the type of health care system may play a role (19, 21, 42). Additionally, future research is required to investigate the rarely examined association between HRQoL and cognitive decline and persistent frequent attendance among the oldest old.

### Strengths and Limitations

Some strengths are worth noting. To our knowledge, this is the first longitudinal study examining the determinants of persistent frequent attendance in primary care among the oldest old. Longitudinal data were taken from a large multicentre prospective cohort study of individuals 85 years and over (including individuals residing in institutionalized settings). Widely established and well-validated tools were used in this study.

Our study also had some limitations. While we cannot rule out the possibility of a recall bias to self-assessment of GP visits, it should be noted that the recall period was small (3 months). Previous research revealed that this bias may be small (43). In our study, we could not control for the entire morbidity profile, e.g., the severity of comorbidity and the individual symptoms burden, which may vary over time. The relationship between changes in these clinical factors and older individuals' behavior in using health services should be investigated in further studies. While the baseline assessment of the AgeCoDe study was nearly representative for older adults living in urban areas in Germany (44), it should be acknowledged that some selection and attrition bias cannot be ruled out in the AgeCoDe/AgeQualiDe study (45). Individuals with severe impairments did not participate in the study. For this reason, generalizing our findings to individuals with very severe cognitive or functional impairments may be rather difficult. Similarly, we cannot generalize our results to older individuals living in rural settings. Lastly, the possibility cannot be dismissed that other factors may exist that contribute to persistent frequent attendance among the oldest old [e.g., neuroticism (46)].

## Conclusions

In conclusion, our study stressed the association between frailty and persistent frequent attendance among the oldest old living in urban areas in Germany. Furthermore, being widowed and having chronic conditions may contribute to persistent frequent attendance. Future research (e.g., based on qualitative approaches) is particularly required to clarify the underlying causes of the widowhood–persistent frequent attendance association.

## Data Availability Statement

Due to ethical restrictions involving patient data, underlying data are available upon request from the Working Group Medical Statistics and IT-Infrastructure. Contact information: BW, wiese.birgitt@mh-hannover.de.

## Ethics Statement

The studies involving human participants were reviewed and approved by the Local Ethic Committees of all participating centers: Ethics Commission of the Medical Association Hamburg, Ethics Commission of the University of Bonn, Medical Ethics Commission II, University of Heidelberg at the University Medical Center of Mannheim, Ethics Commission at the Medical Center of the University of Leipzig, Ethics Commission of the Medical Faculty of the Heinrich-Heine-University Düsseldorf, and Ethics Committee of the TUM School of Medicine, Munich. The patients/participants provided their written informed consent to participate in this study.

## Author Contributions

EB-S and AH made substantial contributions to the conception and design of the study, the analysis and interpretation of data, and drafted the manuscript. H-HK and MS made substantial contributions to the analysis and interpretation of data and drafting of the manuscript. SR-H, HB, SW, ME, AO, MLu, MLö, DW, EM, KH, MW, and WM made substantial contributions to conception and design and critically revised the manuscript. BW made substantial contributions to conception and design, contributed to the analyses and data interpretation, and critically revised the manuscript. JW, AF, and MP carried outpatient assessments, contributed to the interpretation of data, and critically revised the manuscript. All authors read and approved the final manuscript.

## Funding

This publication is a part of the German Research Network on Dementia (KND), the German Research Network on Degenerative Dementia (KNDD; German Study on Aging, Cognition, and Dementia in Primary Care Patients; AgeCoDe), and the Health Service Research Initiative [Study on needs, health service use, costs, and HRQoL in a large sample of oldest-old primary care patients (85+; AgeQualiDe)] and was funded by the German Federal Ministry of Education and Research (grants KND: 01GI0102, 01GI0420, 01GI0422, 01GI0423, 01GI0429, 01GI0431, 01GI0433, 01GI0434; grants KNDD: 01GI0710, 01GI0711, 01GI0712, 01GI0713, 01GI0714, 01GI0715, 01GI0716; grants Health Service Research Initiative: 01GY1322A, 01GY1322B, 01GY1322C, 01GY1322D, 01GY1322E, 01GY1322F, 01GY1322G). The publication was also supported by the study Healthy Aging: Gender-specific trajectories into latest life (AgeDifferent.De) that was funded by the German Federal Ministry of Education and Research (grants 01GL1714A; 01GL1714B; 01GL1714C; 01GL1714D).

## Conflict of Interest

The authors declare that the research was conducted in the absence of any commercial or financial relationships that could be construed as a potential conflict of interest.

## Publisher's Note

All claims expressed in this article are solely those of the authors and do not necessarily represent those of their affiliated organizations, or those of the publisher, the editors and the reviewers. Any product that may be evaluated in this article, or claim that may be made by its manufacturer, is not guaranteed or endorsed by the publisher.
